# Dr. Ira Adelbert Hix

**Published:** 1912-05

**Authors:** 


Dr. Ira Adelbert Hix of Binghamton, a graduate of N. Y. University, 1883, died April 12, 1912.

